# Safe composition levels of transgenic crops assessed via a clinical medicine model

**DOI:** 10.1002/biot.200900217

**Published:** 2010-02

**Authors:** Rod A Herman, Peter N Scherer, Amy M Phillips, Nicholas P Storer, Mark Krieger

**Affiliations:** 1 Dow AgroSciences LLCIndianapolis, IN, USA; 2 Dow AgroSciences LLCKensington, MD, USA

**Keywords:** Safety assessment, Statistics, Substantial equivalence, Transgenic crops

## Abstract

Re-use of this article is permitted in accordance with the Creative Commons Deed, Attribution 2.5, which does not permit commercial exploitation.

Substantial equivalence has become established as a foundation concept in the safety evaluation of transgenic crops. In the case of a food and feed crop, no single variety is considered the standard for safety or nutrition, so the substantial equivalence of transgenic crops is investigated relative to the array of commercial crop varieties with a history of safe consumption. Although used extensively in clinical medicine to compare new generic drugs with brand-name drugs, equivalence limits are shown to be a poor model for comparing transgenic crops with an array of reference crop varieties. We suggest an alternate model, also analogous to that used in clinical medicine, where reference intervals are constructed for a healthy heterogeneous population. Specifically, we advocate the use of distribution-free tolerance intervals calculated across a large amount of publicly available compositional data such as is found in the International Life Sciences Institute Crop Composition Database.

## 1 Introduction

Substantial equivalence has become established as a foundation concept in the safety evaluation of transgenic crops. If the composition (nutrients, antinutrients, etc.) of a transgenic crop is found to be equivalent to that of non-transgenic varieties of the same crop, and those crop varieties are considered safe, then further safety assessment of the transgenic crop can focus solely on the intended modification, usually the expression of a transgenic protein that is novel in that crop [1]. A number of statistical approaches have been used to compare the composition of transgenic crops with their conventional counterparts [2–28], and new methods have recently been suggested [29, 30]. However, the concept of substantial equivalence has been adopted in the area of clinical medicine for a much longer period compared with its relatively short application to the field of transgenic crops, so it seems wise to learn from this experience. Here we discuss how the issue of equivalence has been dealt with in the area of clinical medicine, and suggest an analogous approach for evaluating substantial equivalence for transgenic crops. Specifically, we suggest how reference intervals should be calculated for evaluating the substantial equivalence of new transgenic crops relative to existing crop varieties that have a history of safe use. We use the term crop variety here to encompass both inbred lines and hybrids.

Bioequivalence is a common concept in the field of clinical medicine. It is an approach that is typically applied to the evaluation of new generic drugs. The intent of such bioequivalence studies is to compare the performance and bioavailability of a new generic drug with the performance of a commercially available brand-name drug. Equivalence limits are constructed based on arbitrarily set deviations (*e.g*., ±20% of the performance of the brand-name drug) or the variability in the response observed when the brand-name drug is administered (statistical equivalence limits).The performance of the candidate generic drug is then examined to see if it performs within these equivalence limits [31]. These limits are centered on the average performance of the brand-name drug. This is an appropriate approach because the generic and brand-name drug are expected to have the same average performance.

Although this approach has been suggested for evaluating the substantial equivalence of transgenic crops [29, 30], the aforementioned pharmaceutical situation is fundamentally different from that of transgenic-crop composition comparisons. Unlike the pharmaceutical situation, no single variety of a crop is considered the benchmark for safety or nutrition. Rather, a large number of crop varieties are considered safe and nutritious. Furthermore, different crop varieties often have distinct compositional profiles, so that there is an expectation that any single variety, whether transgenic or not, would have a composition that differs from the average composition across all varieties. Therefore, constructing equivalence limits around the average composition across a number of different crop varieties that are each considered safe is not useful for understanding the safety of an individual variety. In fact, if many crop varieties are used to construct statistical equivalence intervals, then many of the individual varieties used to construct the interval will fall outside of the interval. This clearly illustrates the inappropriateness of this approach for evaluating the safety of transgenic crops.

Another concept that is also widely applied to the area of clinical medicine is a better model for the safety analysis of transgenic crops. It is common in the medical field to test individual patients for the presence of analytes (*e.g.*, disease markers or blood chemistry) and to assess whether such results are normal. As in the previous case, intervals are constructed to use as a frame of reference to judge individual patient results [32]. Such intervals maybe based on previous results with diseased patients, or more commonly, are based on responses from a population of healthy individuals. We generally do not have crop varieties that are considered unsafe, but for a small number of crops and analytes, such varieties exist. For example, unsafe levels of glycoalkaloids in a non-transgenic variety of potatoes led to intoxication upon consumption, as did cyanogenic compound levels in a non-transgenic lima bean variety [6, 33]. For this reason, new potato and lima bean varieties, whether transgenic or non-transgenic, are routinely tested for these compounds before commercial release. However, for most compositional constituents, unsafe levels are not known to exist in food crops. Thus, in the vast majority of cases, each and every crop variety is considered safe. This is analogous to the field of clinical medicine in which a population of healthy individuals may be used to construct intervals for evaluating the test results of an individual patient [32]. Thus we can look to this example to gain insight into how intervals describing a safe/normal population should be constructed.

There are three common types of statistical intervals: confidence, prediction, and tolerance [34]:

1. Confidence limits describe the interval of certainty around a mean value (or mean difference between two groups). In the bioequivalence example above, if a new generic drug was to produce results within the confidence limits for a standard brand-name drug, then one would not conclude that they are different. These limits are sometimes considered equivalence limits, and falling within them is sometimes asserted to demonstrate equivalence [31]. This approach has merit where two treatments are being tested for having equivalent mean responses (or a zero mean difference between treatments) and/or variability.
2. Prediction intervals estimate the probability that a new sample from the same population will fall within the estimated limits. This measure is rarely used for evaluating equivalence.
3. Tolerance limits describe the interval that is expected to contain a certain specified proportion of the population with a specified level of certainty. For example, one can calculate a tolerance interval that is expected to contain at least 99% of the population with 95% certainty. While a confidence interval will approach a zero-width as the sample size increases to infinity (reflecting the true population mean), tolerance limits converge on the values that contain the specified proportion of the population as the sample size increases. Tolerance limits are used in a number of fields, including clinical medicine, to evaluate whether or not a new response is normal for a healthy individual. For example, if a patient is tested for the presence of a cancer marker, results might be compared with tolerance intervals generated from results with a population of healthy individuals [35]. Results outside the specified tolerance interval indicate that further diagnostics should be conducted.

Of these, tolerance intervals are the most ap propriate for evaluating whether or not a transgenic variety is within the normal range for commercial varieties of the same crop.

One concern with calculating reliable intervals, including tolerance intervals, is obtaining a sufficiently large sample size. If too small a sample size is used to generate a tolerance interval, it is likely to contain far more coverage than specified. This is a consequence of the certainty level that is desired, and is similar to confidence intervals that are very wide for small sample sizes. This situation is especially problematic for intervals that are designed to cover a large proportion of the population with a high degree of certainty. In these cases, if the sample size is too low, the tolerance interval will be too wide to be of practical value [36]. The appropriate sample size for constructing tolerance intervals is also affected by the assumed underlying distribution of the data from which it is calculated. For a normal distribution, a minimum of 120 points is recommended for a 95%-coverage, 90%-certainty, tolerance interval [32]. Higher numbers would be needed for a useful 99%-coverage, 95%-certainty, tolerance interval. When estimating high-coverage tolerance intervals, accurately defining the underlying data distribution is especially important, because the intervals will be very sensitive to deviations from the assumed distribution in the tails of the distribution, where few (if any) points are available [37]. An alternative approach is to calculate distribution-free tolerance intervals. Such intervals are robust if an adequate sample size is used, but this approach requires large sample sizes. For example, a minimum of 473 data points are needed for calculating a 99%-coverage, 95%-certainty, distribution-free, tolerance interval [34, 38].

The International Life Sciences Institute (ILSI) has compiled a large database of compositional results for many non-transgenic varieties of a few widely planted crops [39]. This resource provides an opportunity to calculate valid high-coverage tolerance intervals for many compositional components found in these crops. Here we present these tolerance intervals for corn, cotton, and soybean, and discuss the merits of using these intervals to evaluate the substantial equivalence of transgenic crops compared with conventional crop varieties.

## 2 Materials and methods

Results for an array of compositional components found in corn, cotton, and soybean seed samples were downloaded from the ILSI, version 3.0, crop-composition database (http://www.cropcomposition.org). Distribution-free tolerance intervals (99%-coverage, 95%-certainty) were determined for each crop-analyte combination [40] where possible (N ≥473). The sample size, mean, median and range of each dataset were also determined, and the certainty with which this range covers at least 99% of the population was determined using distribution-free methods [40, 41].This latter calculation is equal to the 99%-coverage, distribution-free tolerance interval at the specified certainty. Results are not reported where the sample size was less than 75.The S-PLUS code used to produce these results was as follows {adapted from Hahn and Meeker [34] (http://www.public.iastate.edu/~wqmeeker/stint.html) and Marcot *et al*. [42]}:

TolInt <- function(data,cov.pct,int.conf){
sampsize <-length(data)
# calculate v - the number of items to strip off the
end(s) of the sorted data n <- 0:sampsize
# calculate point at which cumulative binomial
probability exceeds int.conf, based on cov.pct
hit rate
# this is the number of data points we need to
include to obtain int.conf
u1 <- match(T,pbinom(n,sampsize,cov.pct)>
int.conf)-1
# this is the number of data points at which to
set interval ends
v <- sampsize -u1
# sort data
datasort <- sort(data) ## calculate interval
v1 <- ifelse(v%%2==0,v/2,(v/2)-.5)
v2 <-ifelse(v%%2==0,v/2,v1 + 1)
l<-v1
u <- sampsize-v2+1
lower <- ifelse(l<1,datasort[1],datasort[l])
upper <- ifelse(u>sampsize,datasort[samp
size], datasort [u])
ti <- c(lower,upper)
ti.pct <- tol.pct(sampsize,ifelse(v1<1,1,v1),
ifelse(v2<1,sampsize,sampsize-v2+1),cov.pct)
ti.res <- list(“N Keep / N Cut”=c(u1,v),
“Number of Data Points”=length(data),“Data
Range”=range(data),
“Mean”=mean(data),“Median”=median(data),“
Quantiles”=quantile(data,probs=seq(0,1,by=.1)),
“Coverage Level”=cov.pct,“Confidence Level”
=int.conf,
“Order Statistics”=c(v1,sampsize-v2+1),“Toler
ance Interval”=ti,
“Calculated Confidence Level For Tolerance
Interval”=ti.pct,
“Calculated Confidence Level For Data
Range”=tol.pct(sampsize,1,sampsize,cov.pct),
“Confidence Interval For mean”=c(mean(data)-
(qt(int.conf/1,length(data)-1)*sqrt(var(data)/
length(data))),
mean(data)+(qt(int.conf/1,length(data)-1)*sqrt
(var (data)/length (data)))))
return (ti.res)
{

## 3 Results and discussion

### 3.1 Tolerance intervals

Distribution-free tolerance intervals (99%-coverage, 95%-certainty) for various compositional components of corn seed that are available in the ILSI crop-composition database are compiled in Table 1, along with the sample sizes used to construct them. Sample sizes for some components of corn (Table 1) and all components of cotton (Table 2) and soybean (Table 3) were less than the minimum needed to calculate 99%-coverage, 95%-certainty tolerance intervals (N<473). Based on the sample size, distribution-free methods were used to calculate alternative tolerance intervals where the certainty with which the range of the corn, cotton, and soybean data captured 99% of the population. This latter calculation is equal to the 99%-coverage, distribution-free tolerance interval at the specified certainty. For example, the 99%-coverage, 83.4%-certainty distribution-free tolerance interval for ash in soybean seed is 3.89–6.99% dry weight (Table 3, row 1). These intervals should conservatively capture the safe levels of these compositional components in the seeds of these crops, since 100% of commercial corn, cotton, and soybean varieties are considered compositionally safe. The calculation of certainty with which each tolerance intervals captures 99% coverage provides a measure of the robustness of the interval.

**Table 1:** Maize grain composition

Analyte	Category	Sample Size	Units	Meana)	Median	Rangeb)	Certainty of Range Containing ≥99% Coverage	99% Coverage 95% Certainty Tolerance Intervalb)
ash	proximates	1410	% DW	1.44	1.41	0.616–6.280	99.999%%	0.834–4.489
carbohydrate	proximates	1410	% DW	84.65	84.7	77.4–89.5	99.999%	78.4–88.9
crude fat	proximates	260	% DW	3.98	3.94	2.47–5.90	73.417%	not calculable
crud protein	proximates	1434	% DW	10.30	10.21	6.15–17.26	99.999%	6.67–16.86
total fat	proximates	1174	% DW	3.56	3.50	1.74–5.82	99.990%	2.09–5.58
acid detergent fiber	fiber	1350	% DW	4.06	3.8	1.82–11.34	99.998%	2.18–9.33
crude fiber	fiber	301	% DW	2.36	2.4	0.49–3.26	80.384%	not calculable
neutral detergent fiber	fiber	1349	% DW	11.23	10.93	5.59–22.64	99.998%	6.74–19.85
total dietary fiber	fiber	397	% DW	16.43	15.48	8.85–35.31	90.731%	not calculable
calcium	minerals	1344	mg/kg DW	46.35	45.2	12.7–208.4	99.998%	17.7–112.4
copper	minerals	1255	mg/kg DW	1.74	1.65	0.073–18.50	99.995%	0.77–4.12
iron	minerals	1255	mg/kg DW	21.81	21.83	10.42–49.07	99.995%	10.58–39.20
magnesium	minerals	1257	mg/kg DW	1194	1192	594–1940	99.996%	768–1601
manganese	minerals	1256	mg/kg DW	6.18	5.94	1.69–14.3	99.995%	2.06–11.5
phosphorus	minerals	1349	mg/kg DW	3273	3279	1470–5330	99.998%	1632–4359
potassium	minerals	1257	mg/kg DW	3842	3786	1810–6030	99.996%	2240–5354
sodium	minerals	300	mg/kg DW	26.73	5.26	0.17–731.54	80.235%	not calculable
zinc	minerals	1257	mg/kg DW	21.55	21.3	6.5–37.2	99.996%	8.7–33.2
alanine	amino acids	1350	mg/g DW	7.90	7.81	4.39–13.93	99.998%	4.81–12.03
arginine	amino acids	1350	mg/g DW	4.33	4.41	1.19–6.39	99.998%	2.14–6.19
aspartic acid	amino acids	1350	mg/g DW	6.88	6.87	3.35–12.08	99.998%	4.44–10.50
cystine	amino acids	1350	mg/g DW	2.21	2.17	1.25–5.14	99.998%	1.33–3.62
glutamic acid	amino acids	1350	mg/g DW	20.09	19.89	9.65–35.36	99.998%	11.43–31.78
glycine	amino acids	1350	mg/g DW	3.85	3.83	1.84–5.39	99.998%	2.50–5.28
histidine	amino acids	1350	mg/g DW	2.96	2.94	1.37–4.34	99.998%	1.98–4.16
isoleucine	amino acids	1350	mg/g DW	3.68	3.61	1.79–6.92	99.998%	2.32–5.96
leucine	amino acids	1350	mg/g DW	13.41	13.21	6.42–24.92	99.998%	7.42–21.74
lysine	amino acids	1350	mg/g DW	3.15	3.12	1.72–6.68	99.998%	2.18–6.21
methionine	amino acids	1350	mg/g DW	2.09	2.05	1.24–4.68	99.998%	1.3–3.7
phenylalanine	amino acids	1350	mg/g DW	5.25	5.19	2.44–9.30	99.998%	3.07–8.21
proline	amino acids	1350	mg/g DW	9.51	9.49	4.62–16.32	99.998%	5.95–14.21
serine	amino acids	1350	mg/g DW	5.12	5.11	2.35–7.69	99.998%	2.88–7.51
threonine	amino acids	1350	mg/g DW	3.75	3.57	2.24–6.66	99.998%	2.35–6.47
tryptophan	amino acids	1350	mg/g DW	0.627	0.613	0.271–2.150	99.998%	0.406–1.080
tyrosine	amino acids	1350	mg/g DW	3.36	3.37	1.03–6.42	99.998%	1.31–5.64
valine	amino acids	1350	mg/g DW	4.90	4.82	2.66–8.55	99.998%	3.34–7.23
16:0 palmitic	fatty acids	1344	% FA	11.50	11.43	7.94–20.71	99.998%	8.07–16.39
16:1 palmitoleic	fatty acids	596	% FA	0.154	0.149	0.095–0.447	98.242%	0.095–0.447
18:0 stearic	fatty acids	1344	% FA	1.82	1.78	1.02–3.40	99.998%	1.13–3.10
18:1 oleic	fatty acids	1344	% FA	25.81	25.2	17.4–40.2	99.998%	17.9–38.7
18:2 linoleic	fatty acids	1344	% FA	57.63	58.45	36.2–66.5	99.998%	40.8–65.9
18:3 linolenic	fatty acids	1344	% FA	1.20	1.16	0.57–2.25	99.998%	0.72–2.20
20:0 arachidic	fatty acids	988	% FA	0.412	0.4	0.297–0.965	99.947%	0.283–0.816
20:1 eicosenoic	fatty acids	987	% FA	0.297	0.293	0.17–1.92	99.946%	0.183–0.453
22:0 behenic	fatty acids	924	% FA	0.176	0.171	0.11–0.349	99.904%	0.11–0.319
beta-carotene	vitamins	278	mg/100g DW	0.680	0.523	<0.026–4.681	76.704%	not calculable
beta-tocopherol	vitamins	224	mg/100g DW	0.140	<0.06	<0.05–2.28	65.656%	not calculable
delta-tocopherol	vitamins	224	mg/100g DW	0.151	<0.06	<0.048–1.61	65.656%	not calculable
folic acid	vitamins	896	mg/100g DW	0.065	0.066	<0.011–0.146	99.877%	0.147–0.132
gamma-tocopherol	vitamins	367	mg/100g DW	2.95	2.86	0.646–6.100	88.228%	not calculable
total tocopherols	vitamins	278	mg/100g DW	4.04	3.81	0.869–13.30	76.704%	not calculable
vitamin B1 (thiamin)	vitamins	894	mg/100g DW	0.530	0.412	0.126–40000	99.874%	0.138–3.501
vitamin B2 (riboflavin)	vitamins	896	mg/100g DW	0.109	0.111	0.05–0.236	99.877%	<0.1–0.234
vitamin B3	vitamins	415	mg/100g DW	2.38	2.32	1.04–4.69	91.984%	not calculable
vitamin B6	vitamins	415	mg/100g DW	0.644	0.635	0.368–1.132	91.984%	not calculable
vitamin E	vitamins	863	mg/g DW	0.0103	0.0095	0.0015–0.0687	99.834%	0.0018–0.0417
phytic acid	bio-actives	1196	% DW	0.745	0.733	0.111–1.570	99.992%	0.18–1.38
raffinose	bio-actives	743	% DW	0.126	0.123	0.01–0.32	99.514%	0.01–0.29
trypsin inhibitor	bio-actives	702	TIU/mg DW	7.72	2.65	<2–7.18	99.302%	<2–6.73
ferulic acid	other metabolites	817	mg/kg DW	2201	2180	291.9–33860	99.749%	542–3842
furfural	other metabolites	230	mg/kg DW	0.675	0.5	<0.5–6.34	67.065%	not calculable
inositol	other metabolites	505	mg/kg DW	1329	1367	<45–3765	96.188%	<45–3765
p-coumaric acid	other metabolites	817	mg/kg DW	218.4	202	53.4–576.2	99.749%	67.3–551.3

The number of significant figures represented in the table reflects the data available in the ILSI Crop Composition Database.

a) Where data includes values less than the level of quantification (LOQ), values of 1/2 the LOQ were used to calculate means.

b) Where data include values of “<LOQ”, values were ranked based on 1/2 the LOQ for establishing ranges and tolerance intervals, but reported intervals reflect the actual LOQ.

**Table 2:** Cotton seed composition

Analyte	Category	Sample Size	Units	Mean	Median	Range	Certainty of Range Containing ≥99% Coverage	99% Coverage 95% Certainty Tolerance Interval
ash	proximates	164	% DW	4.46	4.55	3.65–5.34	48.892%	not calculable
calories	proximates	156	Kcal/100g DW	474.2	479.5	407.4–508.1	46.297%	not calculable
carbohydrate	proximates	156	% DW	47.35	47.3	39–53.6	46.297%	not calculable
crude protein	proximates	164	% DW	26.88	26.68	21.48–32.97	48.892%	not calculable
total fat	proximates	156	% DW	21.55	21.68	17.2–27.29	46.297%	not calculable
acid detergent fiber	fiber	110	% DW	28.71	29.53	19.74–38.95	30.115%	not calculable
crude fiber	fiber	142	% DW	17.75	17.38	13.86–23.10	41.578%	not calculable
neutral detergent fiber	fiber	110	% DW	39.65	40.53	25.56–51.87	30.115%	not calculable
total dietary fiber	fiber	71	% DW	41.76	42.57	33.69–47.55	47.550%	not calculable
calcium	minerals	150	mg/kg DW	1472	1475	1032–3258	44.302%	not calculable
copper	minerals	150	mg/kg DW	7.54	7.59	3.13–24.57	44.302%	not calculable
iron	minerals	150	mg/kg DW	53.78	51.79	36.71–318.38	44.302%	not calculable
magnesium	minerals	150	mg/kg DW	4138	4127	3471–4931	44.302%	not calculable
manganese	minerals	150	mg/kg DW	15.32	14.87	10.69–21.96	44.302%	not calculable
phosphorus	minerals	150	mg/kg DW	7256	7278	4825–9916	44.302%	not calculable
potassium	minerals	150	mg/kg DW	11886	11890	9835–14484	44.302%	not calculable
sodium	minerals	145	mg/kg DW	1269	1150	110.7–7355.	42.607%	not calculable
zinc	minerals	150	mg/kg DW	37.96	37.7	27–59.5	44.302%	not calculable
alanine	amino acids	149	mg/g DW	10.17	10.14	8.01–12.19	43.965%	not calculable
arginine	amino acids	149	mg/g DW	28.28	28.14	20.57–37.22	43.965%	not calculable
aspartic acid	amino acids	141	mg/g DW	23.37	23.40	18.25–27.48	41.232%	not calculable
cystine	amino acids	149	mg/g DW	4.36	4.38	3.47–5.57	43.965%	not calculable
glutamic acid	amino acids	149	mg/g DW	51.10	50.96	39.14–67.21	43.965%	not calculable
glycine	amino acids	149	mg/g DW	10.65	10.63	8.31–13.16	43.965%	not calculable
histidine	amino acids	149	mg/g DW	7.36	7.33	5.73–9.06	43.965%	not calculable
isoleucine	amino acids	149	mg/g DW	8.23	5.22	6.20–10.46	43.965%	not calculable
leucine	amino acids	149	mg/g DW	15.04	14.96	11.39–18.55	43.965%	not calculable
lysine	amino acids	149	mg/g DW	11.88	11.75	9.41–14.56	43.965%	not calculable
methionine	amino acids	149	mg/g DW	3.89	3.90	3.02–4.69	43.965%	not calculable
phenylalanine	amino acids	149	mg/g DW	13.45	13.39	10.19–17.15	43.965%	not calculable
proline	amino acids	149	mg/g DW	9.85	9.77	7.53–12.30	43.965%	not calculable
serine	amino acids	149	mg/g DW	11.53	11.59	9.15–13.51	43.965%	not calculable
threonine	amino acids	149	mg/g DW	7.78	7.86	5.53–9.18	43.965%	not calculable
tryptophan	amino acids	149	mg/g DW	2.59	2.55	1.94–3.19	43.965%	not calculable
tyrosine	amino acids	149	mg/g DW	6.67	6.64	5.25–8.41	43.965%	not calculable
valine	amino acids	149	mg/g DW	11.54	11.49	8.67–14.89	43.965%	not calculable
14:0 myristic	fatty acids	150	% FA	0.822	0.798	0.455–2.400	44.302%	not calculable
16:0 palmitic	fatty acids	150	% FA	23.50	23.66	15.11–27.90	44.302%	not calculable
16:1 palmitoleic	fatty acids	149	% FA	0.617	0.607	0.451–1.190	43.965%	not calculable
18:0 stearic	fatty acids	150	% FA	2.43	2.41	0.2–3.11	44.302%	not calculable
18:1 oleic	fatty acids	150	% FA	16.41	16.55	12.8–25.3	44.302%	not calculable
18:2 linoleic	fatty acids	150	% FA	54.26	54.4	46–59.4	44.302%	not calculable
18:3 linolenic	fatty acids	77	% FA	0.204	0.19	0.11–0.42	18.005%	not calculable
20:0 arachidic	fatty acids	150	% FA	0.272	0.271	0.186–0.414	44.302%	not calculable
22:0 behenic	fatty acids	147	% FA	0.150	0.145	0.104–0.295	43.288%	not calculable
dihydrosterculic	fatty acids	145	% FA	0.179	0.174	0.075–0.310	42.607%	not calculable
malvalic	fatty acids	150	% FA	0.419	0.419	0.229–0.759	44.302%	not calculable
sterculic	fatty acids	150	% FA	0.297	0.292	0.19–0.556	44.302%	not calculable
free gossypol	bio-actives	155	% DW	0.802	0.765	0.454–1.399	45.968%	not calculable
total gosspol	bio-actives	164	% DW	0.966	0.942	0.547–1.522	48.892%	not calculable

The number of significant figures represented in the table reflects the data available in the ILSI Crop Composition Database.

**Table 3:** Soybean seed composition

Analyte	Category	Sample Size	Units	Mean	Median	Range	Certainty of Range Containing ≥99% Coverage	99% Coverage 95% Certainty Tolerance Interval
ash	proximates	323	% DW	5.32	5.29	3.89–6.99	83.410%	not calculable
carbohydrate	proximates	323	% DW	38.24	37.80	29.60–50.20	83.410%	not calculable
crude protein	proximates	323	% DW	39.47	39.33	33.19–45.48	83.410%	not calculable
total fat	proximates	323	% DW	16.68	17.25	8.10–23.56	83.410%	not calculable
acid detergent fiber	fiber	149	% DW	11.97	11.78	7.81–18.61	43.965%	not calculable
crude fiber	fiber	234	% DW	7.81	7.82	4.12–13.87	67.978%	not calculable
neutral detergent fiber	fiber	149	% DW	12.33	12.11	8.53–21.25	43.965%	not calculable
calcium	minerals	80	mg/kg DW	2171	2068	1166–3071	19.084%	not calculable
iron	minerals	80	mg/kg DW	78.11	77.45	55.36–109.54	19.084%	not calculable
magnesium	minerals	80	mg/kg DW	2636	2591	2194–3128	19.084%	not calculable
phosphorus	minerals	80	mg/kg DW	7148	7394	5067–9352	19.084%	not calculable
potassium	minerals	80	mg/kg DW	20614	20621	18680–23161	19.084%	not calculable
alanine	amino acids	234	mg/g DW	17.16	17.10	15.13–21.04	67.978%	not calculable
arginine	amino acids	234	mg/g DW	28.40	27.92	22.85–34.00	67.978%	not calculable
aspartic acid	amino acids	234	mg/g DW	44.93	44.57	38.08–51.22	67.978%	not calculable
cystine	amino acids	234	mg/g DW	5.87	5.86	3.70–8.08	67.978%	not calculable
glutamic acid	amino acids	234	mg/g DW	70.88	70.20	58.43–82.01	67.978%	not calculable
glycine	amino acids	234	mg/g DW	16.88	16.80	14.58–19.97	67.978%	not calculable
histidine	amino acids	234	mg/g DW	10.40	10.38	8.78–11.75	67.978%	not calculable
isoleucine	amino acids	234	mg/g DW	18.08	18.09	15.39–20.77	67.978%	not calculable
leucine	amino acids	234	mg/g DW	30.39	30.16	25.90–36.22	67.978%	not calculable
lysine	amino acids	234	mg/g DW	25.57	25.51	22.85–28.39	67.978%	not calculable
methionine	amino acids	234	mg/g DW	5.51	5.50	4.31–6.81	67.978%	not calculable
phenylalanine	amino acids	234	mg/g DW	19.79	19.72	16.32–23.46	67.978%	not calculable
proline	amino acids	234	mg/g DW	20.01	19.99	16.87–22.84	67.978%	not calculable
serine	amino acids	234	mg/g DW	20.19	20.12	11.06–24.84	67.978%	not calculable
threonine	amino acids	234	mg/g DW	14.73	14.56	11.39–18.62	67.978%	not calculable
tryptophan	amino acids	234	mg/g DW	4.33	4.32	3.56–5.02	67.978%	not calculable
tyrosine	amino acids	234	mg/g DW	3.21	13.12	10.16–16.13	67.978%	not calculable
valine	amino acids	234	mg/g DW	19.10	19.20	15.97–22.04	67.978%	not calculable
16:0 palmitic	fatty acids	234	% FA	11.12	10.97	9.55–15.77	67.978%	not calculable
16:1 palmitoleic	fatty acids	122	% FA	0.127	0.123	0.086–0.194	34.499%	not calculable
17:0 heptadecanoic	fatty acids	97	% FA	0.114	0.115	0.085–0.146	25.315%	not calculable
18:0 stearic	fatty acids	234	% FA	4.01	3.98	2.70–5.88	67.978%	not calculable
18:1 oleic	fatty acids	234	% FA	20.72	20.60	14.30–32.20	67.978%	not calculable
18:2 linoleic	fatty acids	234	% FA	53.26	53.40	42.30–58.80	67.978%	not calculable
18:3 linolenic	fatty acids	234	% FA	8.34	8.21	3.00–12.52	67.978%	not calculable
20:0 arachidic	fatty acids	233	% FA	0.323	0.319	0.163–0.482	67.752%	not calculable
20:1 eicosenoic	fatty acids	221	% FA	0.204	0.192	0.14–0.35	64.934%	not calculable
22:0 behenic	fatty acids	233	% FA	0.402	0.391	0.277–0.595	67.752%	not calculable
folic acid	vitamins	80	mg/100g DW	0.359	0.376	0.239–0.471	19.084%	not calculable
vitamin B1 (thiamin)	vitamins	80	mg/100g DW	0.197	0.197	0.101–0.254	19.084%	not calculable
vitamin B2 (riboflavin)	vitamins	80	mg/100g DW	0.267	0.272	0.19–0.321	19.084%	not calculable
vitamin E	vitamins	234	mg/g DW	0.0191	0.0135	0.0019–0.0617	67.978%	not calculable
lectins	bio-actives	251	H.U./mg DW	1.72	1.27	0.11–9.04	71.630%	not calculable
phytic acid	bio-actives	118	% DW	1.12	1.13	0.63–1.96	33.046%	not calculable
raffinose	bio-actives	118	% DW	0.35	0.34	0.21–0.66	33.046%	not calculable
stachyose	bio-actives	118	% DW	2.19	2.23	1.21–3.50	33.046%	not calculable
total diadzein	bio-actives	289	mg/kg DW	862.6	784.0	60.00–2453	78.533%	not calculable
total genitein	bio-actives	289	mg/kg DW	978.6	893.8	144.3–2837.	78.533%	not calculable
total glycitein	bio-actives	286	mg/kg DW	161.2	160.2	15.30–310.4	78.047%	not calculable
total isoflavones	bio-actives	76	mg/kg DW	2221	2006	678.7–3733.	17.647%	not calculable
trypsin inhibitor	bio-actives	178	TIU/mg DW	48.33	45.99	19.59–118.68	53.236%	not calculable

The number of significant figures represented in the table reflects the data available in the ILSI Crop Composition Database.

As described earlier, the approach of using tolerance intervals to describe the range of response variables expected when testing a healthy population has precedence in the area of clinical medicine [32, 35, 43]. This approach has also been used to compare the compositional and nutritional equivalence of transgenic crops with populations of non-transgenic crops [9, 10, 12, 19, 21, 24, 25, 35, 39, 44, 45]. However, large sample sizes are required to calculate tolerance intervals that are not so wide as to be of little practical value [36]. Furthermore, the construction of tolerance intervals is very sensitive to deviations from the assumed distribution especially for high-coverage, high-certainty intervals like those typically constructed [37]. For this reason, we used the publicly available data in the ILSI crop-composition database to construct useful 99%-coverage, 95%-certainty, distribution-free, tolerance intervals where possible (N ≥473). In cases where the sample size was insufficient to calculate 99%-coverage tolerance intervals with 95% certainty, the certainty with which the range of the data covers at least 99% of the population was calculated.

We used all data for each analyte in the ILSI crop-composition database and did not segregate the data by the analytical method used to determine the compositional component, or by the laboratory used to analyze the samples. Only validated methods that are comparable should be used to determine the composition of crops if such data are to be used for a safety assessment. This validation process should include spike-recovery experiments that demonstrate that the method is able to recover an adequate proportion of the analyte, and each laboratory should validate its ability to carry out the analyses successfully. The methods used to determine compositional analytes compiled in the ILSI crop-composition database are accepted, validated, and independently-developed [39]. Furthermore, the acceptance criteria required by ILSI for including data are stringent and potential outliers are confirmed as valid before entry into the database [39]. As such, all data in the database, regardless of the analytical method, should be comparable.

However, some data in the ILSI crop composition database may display a bimodal distribution correlated with the method of analysis (*e.g.*, vitamin B1). In these cases, we recommend that single homogeneous subsamples of plant tissue be sent to the different laboratories conducting the analyses in question, along with additional subsamples that have been fortified with a well-characterized purified preparation of the analyte in question. The characterization of the purified standard should include an absolute purity estimate based on the best methods available, and if possible, be verified by an additional analytical method. If the laboratories obtain equivalent results for these samples, then the distribution of values in the database may represent true differences in the analyte concentrations between the germplasm sources sent to each laboratory. If the laboratories obtain different results for the subsamples sent to each laboratory, then it will be possible to subtract the results for the non-fortified sample from those of the fortified sample and determine the accuracy of each laboratory or method. Finally, if the accuracy of the laboratories for predicting the correct quantity of fortification is good for both laboratories, but results from the unfortified samples differ, the laboratory with the lower results for the non-fortified samples may have inferior extraction methods. Since the units associated with analytes in the database are absolute, meaningfully inaccurate results should be removed from the database, or the units changed to an index scale. It is also important to be sure that subtle differences in the actual analyte being measured are not causing differences. If this is the case, then more explicit analyte names should replace the current names to more clearly segregate the data.

We also included all geographies and growing seasons in our datasets, since the compositional safety of corn, cotton, and soybean is not known to be compromised when these crops are grown in any geography or environment, whether consumed locally or in other regions. This differs from the model sometimes used in clinical medicine where tolerance limits may be generated regionally, because results for a healthy subpopulation in one region may indicate disease in another subpopulation in a different region. As indicated above, this does not apply to crop varieties that are safely grown and consumed worldwide.

In addition to the advantages previously described for distribution-free tolerance intervals, a couple of additional points are worthy of mention. Many of the data in the ILSI crop-composition database were collected from replicated field trials like those used to evaluate transgenic lines. Therefore, any bias in the sampling of such data should be roughly equivalent between these two groups of data, making the data distributions similar between groups. However, it must be acknowledged that, like previous studies in the clinical field and those used to evaluate the substantial equivalence of transgenic crops in the past [9, 10, 12, 19, 21, 24, 25, 35, 39, 44, 45], sample results may not represent truly random independent samples, and correlations within the samples likely exist, theoretically reducing calculated tolerance intervals such that they do not span the designated coverage. As such, the tolerance intervals reported here may be more conservative than those generated from truly random samples. Furthermore, it is important to investigate the distribution of samples collected from specific field trials when comparing them with the tolerance intervals reported here to check the assumption that both datasets appear to be distributed similarly.

An addition advantage of tolerance intervals is simplicity of interpretation. It is easy to understand the coverage encompassed in tolerance intervals and the degree of certainty that one has about this content. The ability to work in the natural units of analyte concentration, as opposed to transformed units that might be applied in an attempt to normalize datasets for a parametric analysis, also simplifies interpretation of results. Concentrations of analytes can be directly compared with literature pertinent to their safety or nutrition without back-calculation of transformed values. Finally, data indicating analyte concentrations below the level of detection or quantification do not need to be censored or assigned “dummy” values for reporting, because distribution-free tolerance intervals are based on the rank of responses, not the responses themselves. This further simplifies analysis and interpretation of results.

Here we have applied techniques analogous to those used in the field of clinical medicine to estimate the normal range of analytes in several non-transgenic crops. The concept of substantial equivalence has been used in the field of clinical medicine for much longer than its fairly recent application to transgenic crops, so it seems natural to make use of the progress made in this area. However, it is noteworthy that tolerance intervals are used in the medical field because it is not possible to ensure that the reference population contains 100% healthy individuals. By using a limited-coverage tolerance interval that excludes a small proportion of subjects, potentially diseased individuals are excluded from what is considered normal. For many crops, such as corn, cotton, and soybean, no unsafe crop varieties are known. As such, the use of 99%-coverage, tolerance intervals to assess safe levels of crop components may be unnecessarily conservative. By way of example, if the US produces 10 billion bushels of corn in any given year and 1% of this crop is considered to be of questionable safety based on composition, this would result in the production of 100 million bushels of potentially unsafe corn in the US every year. Unless invalid data are present in the reference database, all determined levels should be safe, and the range of the data is an appropriately conservative interval to use for assessing safety.

In reality, the range of the data in the ILSI database may also be too conservative, because many varieties are not represented in this database, and studies with some non-transgenic varieties indicate that their composition is frequently outside of this range [23]. In addition, the sample sizes for many crop-analyte combinations are not sufficiently robust to capture an adequate cross-section of the expected variability across all varieties. For these reasons, compositional equivalence studies typically include a concurrently grown non-transgenic, near-isogenic line, and sometimes include various other commercial reference lines. Such lines can be used to supplement the range of responses found in the ILSI database. The composition of samples collected from transgenic varieties can be compared with intervals constructed from the values tabulated in the ILSI crop-composition database and from these concurrently grown reference lines to evaluate substantial equivalence. Traditional analysis of variance approaches comparing concurrently grown controls with transgenic lines may also be useful in assessing whether or not varietal differences are statistically significant. Finally the safety consequences of any differences will need to be assessed in the context of biological impact. It is important to understand that compositional equivalence studies with transgenic crops are typically conducted to inform the safety assessment, and are not conducted to detect minor changes from the non-transgenic isogenic line. While such changes may be of academic interest, they do not suggest a safety risk if compositional components are within the normal range for a crop that is safely consumed regardless of the variety. It is also noteworthy that perfect isogenic lines are never actually available because native genes closely linked to the transgenic traits will always be present in the transgenic line at higher frequencies than in the non-transgenic isogenic control, and these genes will likely result in some compositional differences between the transgenic line and the non-transgenic isogenic line. However, this phenomenon is likely more dramatic when polygenic traits are selected in traditional breeding programs with non-transgenic crops.

### 3.2 Value of compositional analyses in safety assessment

It is unclear how the insertion of a novel gene would disrupt the genome causing an unsafe perturbation of composition in a fundamentally different manner than that experienced during traditional breeding or mutagenesis, and the current literature supports the concept that agronomically acceptable varieties containing transgenic insect-resistance genes and herbicide-tolerance genes are not particularly prone to such changes [2–28]. A long history of crop improvement, resulting in very few adverse health effects, suggests that our current food crops are not generally prone to up-regulation of detrimental constituents. In fact, this attribute of these plant species likely contributed to their selection and persistence as food crops. Furthermore, agronomically “off-types” are culled from any breeding program, whether traditional or transgenic. Compositional analysis is, none the less, required by most governments for approval of transgenic plants, but not for non-transgenic crop varieties.

Regulation of non-transgenic crop composition was attempted in the early 1970s when the FDA enacted similar but much less aggressive regulation in the area of traditional crop breeding [46]. However, the regulations were impractical and unenforceable, and today, have been largely forgotten. In addition, novel food regulations are in place in several geographies, but these do not generally extend to new non-transgenic varieties of food crops unless expressly bred to have an altered composition [47].

Compositional equivalence studies have added little to the safety assessment of currently available transgenic crops since unsafe levels of compositional components have not been identified [2–28]. However, new transgenic crops are in development that are expressly intended to have modified composition. While the likelihood of altering the safety of transgenic crops through DNA-insertional effects may be lower than for traditional breeding [16, 18, 20, 22, 39], the safety assessment for new transgenic crops bearing traits intended to alter endogenous metabolic pathways may be aided by hypothesis-driven compositional analyses.

## 4 Concluding remarks

The use of tolerance intervals using appropriate sample sizes, and covering many varieties and environments, represents a valid statistical approach for assessing the composition of transgenic crops in relation to their conventional counterparts. For crops that are not known to contain unsafe levels of compositional components, the range of compositional data for commercially available varieties is an adequately conservative safety interval. This approach has the most value for assessing the safety of traits intended to alter endogenous metabolic pathways in plants, but compositional analysis for input traits is generally not warranted. To support these methods, the continued submission of quality data to the ILSI crop composition database is strongly encouraged, especially where the sample sizes are insufficient to calculate distribution-free 99%-coverage 95%-certainty tolerance intervals (N < 473).

Statistical approaches to data analysis are almost universally required when reporting data to regulatory agencies or in peer-reviewed journals. Here we describe the application of a statistical approach used in clinical medicine to the evaluation of substantial equivalence of transgenic crops and non-transgenic crops, and suggest that the greater experience in the field of clinical medicine should make this model the standard against which other approaches are compared. We describe the methods used to construct 99%-coverage, 95%-certainty tolerance intervals, and also how to determine the certainty that the range of data for non-transgenic crops covers 99% of the data. Both types of tolerance intervals should be useful in complying with the need to present statistical measures of compositional equivalency to support the safety assessment of transgenic crops, and Tables 1–3 should be a handy resource for comparing the composition of new transgenic corn, soybean, and cotton varieties with conventional comparators. While beyond the scope of this publication, the methods and intervals reported here can be compared with those reported elsewhere using alternative methods.
